# Clinical outcomes and safety assessment in elderly patients undergoing decompressive laminectomy for lumbar spinal stenosis: a prospective study

**DOI:** 10.1186/1471-2482-10-34

**Published:** 2010-11-22

**Authors:** Asgeir S Jakola, Andreas Sørlie, Sasha Gulati, Øystein P Nygaard, Stian Lydersen, Tore Solberg

**Affiliations:** 1 Department of Neurosurgery, St. Olavs Hospital HF, N-7006 Trondheim, Norway; 2 Department of Circulation and Medical Imaging, Norwegian University of Science and Technology, N-7005, Trondheim, Norway; 3 Department of Neurosurgery, University Hospital of North Norway, N-9038 Tromsø, Norway; 4 The Norwegian National Registry for Spine Surgery, SKDE, North Norway Regional Health Authority, Tromsø, Norway; 5 Department of laboratory medicine, Children's and Women's Health, Norwegian University of Science and Technology, Trondheim, Norway; 6 National Center for Spinal Disorders, Faculty of Medicine, Norwegian University of Science and Technology, Trondheim, Norway; 7 Department for Applied Clinical Research, Children's and Women's Health, Norwegian University of Science and Technology, Trondheim, Norway; 8 Department of Neuroscience, Norwegian University of Science and Technology, N-7005, Trondheim, Norway

## Abstract

**Background:**

To assess safety, risk factors and clinical outcomes in elderly patients with spinal stenosis after decompressive laminectomy.

**Methods:**

A prospective cohort of patients 70 years and older with spinal stenosis undergoing conventional laminectomy without fusion (n = 101) were consecutively enrolled from regular clinical practice and reassessed at 3 and 12 months. Primary outcome was change in health related quality of life measured (HRQL) with EuroQol-5 D (EQ-5D). Secondary outcomes were safety assessment, changes in Oswestry disability index (ODI), Visual Analogue Scale (EQ-VAS) score for self reported health, VAS score for leg and back pain and patient satisfaction. We used regression analyses to evaluate risk factors for less improvement.

**Results:**

The mean EQ-5 D total score were 0.32, 0.63 and 0.60 at baseline, 3 months and 12 months respectively, and represents a statistically significant (P < 0.001) improvement. Effect size was > 0.8. Mean ODI score at baseline was 44.2, at 3 months 25.6 and at 27.9. This represents an improvement for all post-operative scores. A total of 18 (18.0%) complications were registered with 6 (6.0%) classified as major, including one perioperative death. Patients stating that the surgery had been beneficial at 3 months was 82 (89.1%) and at 12 months 73 (86.9%). The only predictor found was patients with longer duration of leg pain had less improvement in ODI (P < 0.001). Increased age or having complications did not predict a worse outcome in any of the outcome variables.

**Conclusions:**

Properly selected patients of 70 years and older can expect a clinical meaningful improvement of HRQL, functional status and pain after open laminectomy without fusion. The treatment seems to be safe. However, patients with longstanding leg-pain prior to operation are less likely to improve one year after surgery.

## Background

Lumbar spinal stenosis (LSS) is the most frequent indication for spinal surgery in the elderly [1,2]. Decompressive laminectomy without fusion is the most commonly used surgical treatment, but the surgical risk is higher in the elderly population due to higher co-morbidity [1,3,4]. Furthermore, patients with co-morbidity are less satisfied with the results of surgery [5]. However, reports on complication rates among elderly patients undergoing laminectomy are conflicting [1,4,6-9]. Indication for surgery most often is relative, and improvement in pain, functional status and quality of life are the main treatment goals. An expectant or non surgical approach is a good option for patients with moderate symptoms and sparse motivation, or when the risk of treatment outweighs potential benefits [6,10,11]. In clinical decision making, both patients and clinicians will have to consider outcomes and risk factors associated with the treatment given.

The aim of this study was to provide information about safety, risk factors and potential benefits of the surgical treatment in an unselected, aged cohort, recruited from daily clinical practice. We therefore evaluate complication rates, changes in quality of life, functional status and pain among elderly patients operated with open laminectomy for lumbar spinal stenosis.

## Methods

### Patient selection

In this prospective study, all patients (n = 101) were enrolled consecutively between 2000 and 2006 from regular clinical practice at a single institution (University Hospital of Northern Norway, UNN, Department of Neurosurgery). All patients were included, and 100 out of 101 completed at least one follow up appointment. Patients 70 years and older with isolated spinal stenosis treated with conventional decompressive laminectomy were included in this study. We define a conventional laminectomy as an operation performed through a midline incision, with removal of the spinous process and lamina. Medial facetectomy was performed when necessary. When decompressing the root canal, the surgical guideline was to preserve as much as possible of the facet joint. Backpain was no exclusion criteria except among patients with radiological signs of instability (spondylolisthesis) who where considered candidates for fusion procedures. Extirpation of herniated discs was done in cases where disc bulging or herniation contributed to stenosis. The operations were performed by specialists (n = 6) and residents (n = 4) in neurosurgery. Of patients 70 years and older with spinal stenosis admitted for surgery we excluded in total 7 patients in this study. In 3 patients there was radiological evidence of instability and they were treated with a fusion procedure. The other 4 patients had isolated spinal stenosis, but were treated with an interspinous distraction device. No patients were excluded because of previous medical history or spine surgery. Follow up time was 12 months from the date of operation (baseline).

### Patient population

Baseline parameters and inpatient data are presented in table 1. Mean age was 75.3 years and 50 (49.5%) were females. The indications were isolated central spinal stenosis in 79 (78.2%) of the cases and both central and lateral (recess) stenosis in 16 (15.8%). Median duration of back pain was 100 (range 0-2020) weeks and median duration of leg pain was 92 (range 0-624) weeks. Median hospital stay was 6 days. One-level laminectomy was performed in 48 (48.0%), two-levels in 42 (42.0%), three-levels in 9 (9.0%) and four-levels in 1 (1.0%) of the cases. Hence, 52 patients (52.0%) underwent multi-level laminectomy. Twenty-two (21.8%) had undergone back surgery previously including 7 (6.9%) patients who were registered more than once due to re-operations after laminectomy for spinal stenosis in the study period. The reasons for re-operations in the 7 patients were spinal stenosis at another level in 4, stenosis at the same level in 2 and recess stenosis at the same level in 1. Of these patients, 2 (2.0%) were re-operated within 12 months of the primary operation. When patients were registered two times (n = 7) during the registration period of 6 years they were considered as re-operations and we used baseline data from the re-operation and outcome data after the re-operation. This was chosen over data from the index operation because we believe it more precisely report patients' final outcome and allowed for inclusion of all re-operated patients in the risk factor analyses.

**Table 1:** Patients' characteristics

Groups Categories	**Total **_†_ No. (%)	**Mean ± SD**_‡ _**(range)**
**Age**	101	75.3 ± 4.1 (70-86)
**Under 76**	57 (56.4)	
**76 and above**	44 (43.6)	
**Sex**	101	
**Male**	51 (50.5)	
**Female**	50 (49.5)	
Duration of back pain * (weeks)	100	100 ± 295 (0-2020)
Duration of leg pain * (weeks)	100	92 ± 132 (0 - 624)
**ASA score**	101	
**1**	7 (6.9)	
**2**	65 (64.4)	
**3**	28 (27.7)	
**4**	1 (1.0)	
**Smoking**	100	
**Yes**	14 (14.0)	
**No**	86 (86.0)	
**Body mass index**	96	26.8 ± 3.1 (18.8-33.7)
**< 20**	1 (1.0)	
**20-24.9**	29 (30.2)	
**25-29.9**	50 (52.1)	
**30 +**	16 (16.7)	
Hospital stay (days)*	101	6 ± 3 (2-15)
**Levels of laminectomy**	100	
**1 level**	48 (48.0)	
**> 1 level**	52 (52.0)	
**Marital status**	101	
**Cohabiting**	61 (60.4)	
**Live alone**	40 (39.6)	
**Educational level **_§_	99	
**Lower**	88 (88.9)	
**Higher**	11 (11.1)	
**Indication for surgery**	101	
**Central spinal stenosis**	79 (78.2)	
**Lateral spinal stenosis**	6 (5.9)	
**Combination of above**	16 (15.8)	
**Previous back surgery**	101	
**Yes**	22 (21.8)	
**No**	79 (78.2)	

*Central tendency presented as median. _§ _Higher educational level refer to university or similar college of higher learning. _† _Where total of patients is not N = 101 it represents missing values. _‡ _SD; standard deviation.

### Data collection

Data were collected according to the standard protocol of a comprehensive clinical registry for quality control and research. Less than two percent of the patients being operated were not recorded in the registry database [12]. The registry database was linked to the National Population Registry of Norway via the national 11-digit personal identification number. In this way we obtained continuously upgraded information about changing home addresses and dates of death within the study population. Causes of death were available from the medical records. The patients completed self-administered questionnaires at admission. The baseline questionnaire included additional questions about demographics and lifestyle issues.

During the hospital stay, the doctor, responsible for each patient, recorded data concerning diagnosis, treatment and employment status and duration of symptoms according to a standard registration form. Finally all questionnaires and forms were collected and checked for completeness by a dedicated research nurse. The American Society of Anaesthetists grading system (ASA grade I - V) was registered for each patient by a doctor or a specialized nurse from the Department of Anaesthesiology.

### Follow up data

Patients were summoned for follow up visits at 3 and 12 months at the outpatient clinic. Questionnaires were distributed by mail and were completed at home by the patients. At the follow up visits a research nurse collected and checked all questionnaires and interviewed the patients about complications. Patients who did not attend the follow up visits were contacted by the research nurse, and could either make a new appointment for a follow up visit, or complete and return the questionnaire by mail. There were 6 (5.9%) patients at 3 months and 19 (18.8%) patients at 12 months who did not respond. Only one out of 101 patients did not respond at all and was lost to follow up.

### Outcomes

The questionnaires completed by the patients at baseline and follow up were identical, and were used for outcome assessments. Primary outcome was changes in EQ-5 D score. Secondary outcomes were changes in ODI, EQ-VAS and VAS score for leg and back pain from baseline to 3 and 12 months. Patients were also asked to rate perceived benefits of the operation.

#### The EuroQol 5D

EQ-5 D is a generic and preference-weighted measure of health-related quality of life. It evaluates five dimensions: mobility, self-care, activities of daily life, pain and anxiety and/or depression with 3 possible answers to each dimension. This results in the 243 different possible health states which are transformed into an index value. We used the value set based on the main survey from the EuroQol group [13], which has been validated for this patient population [12]. Total score range is from -0.594 to 1, where 1 corresponds to perfect health, and 0 to death. Negative values are considered to be worse than death.

#### Oswestry Disability Index

Oswestry disability index is a scale from 0 to 100 where higher number indicates more severe symptoms [14]. The questionnaire focuses on how patients relate to 10 common activities where different statements about functional level are given. Patients were given a validated Norwegian form of the questionnaire [15].

#### The EuroQol Visual Analogue Scale

The EQ-VAS forms the second part of the EuroQol questionnaire. Patients rated their current health state on a line, which ranges from 0-100 (worst to best imaginable health) [13].

#### Visual analogue scale for rating back and leg pain

Patients marked their current symptoms on symptom specific VAS for back and leg pain. The scale is an unmarked line of 100 mm where end-points referred to 'no symptoms' and 'worst imaginable symptoms'.

#### Benefits of the operation

At follow up (from year 2000 to 2004), the patients were asked: "How much benefit have you had from the operation?" The response alternatives were: "Very much", "Quite a lot", "Some", "None at all" or "Uncertain". They were changed in the registry database after 2003 into: "Very much", "Some", "None at all" or "Worse than before". This change, with the inclusion of a possibility for deterioration after surgery, was done to make the response alternatives more balanced. Because of these changes, we dichotomized all the responses alternatives into "beneficial", including all positive answers and "not beneficial", including neutral and negative point of views in the analyses.

#### Walking capability

To assess walking capability we calculated the median score of the fourth item of the Oswestry Disability Index at baseline and follow up. The response alternatives (0-5) were: no limitations(0), able to walk up to 1,5 kilometers (1), up to 750 meters (2), up to 100 meters (3), dependent of rollator or crutches (4), bedridden (5).

#### Complications

To assess safety we included all complications detected during interviews at 3 and 12 months. Complications were considered major where they may have contributed to death, worsened health condition, readmission to hospital or prolonged hospital stay. All deaths within the study population were registered and investigated.

### Statistics

All analyses were done with the SPSS, version 15.0 (Chicago, IL). Statistical significance level was set to P ≤ 0.05. To test if data were normally distributed we used Q-Q plots. Central tendency are presented as means when normally distributed and as medians when skewed. Comparisons of means were analyzed with paired-samples t-test (two-sided). Where two analyses are performed (at 3 and 12 months), P-values between 0.025 - 0.05 should be interpreted with caution due to increased chance of type 1 error. To compare groups we used independent samples t-test (two-sided). To evaluate the magnitude of change in EQ-5 D score we calculated the effect size (ES), and considered values > 0.8 to represent a large clinical effect [16].

#### Risk factor analyses

To assess possible predictors of outcome we used logistic regression for dichotomous dependent variables and linear regression when data were continuous and normally distributed. Regression analyses were done for all primary and secondary outcome variables plus length of hospital stay. As possible predictors we analyzed the effect of age, sex, smoking status, levels of laminectomy (1 level or more), previous back surgery (yes/no), complications to surgery (yes/no), body mass index (BMI), duration of back and leg pain and ASA score (dichotomous; ASA 1 and 2 versus ASA 3 and 4). The analyses were only done for outcomes at one year. This means that 80 analyses were done and the significance level was therefore adjusted to P < 0.001.

In addition we assessed if outcomes were different between the oldest patients of our cohort (median age and above) and the youngest (below median age), the age variable was dichotomized accordingly.

The Data Inspectorate in Norway approved registration and management of data. The study was approved by the Regional Ethical Committee for health region North Norway.

## Results

### Primary outcome

The mean EQ-5 D total score was 0.32 at baseline, 0.63 at 3 months and 0.60 at 12 months (table 2). The scores were statistically significantly improved by surgery (P < 0.001) at follow up. Changes in EQ-5 D total score represent a large clinical change with ES 0.97 and 0.87 at 3 and 12 months respectively. Changes in each dimension was significant (P < 0.001 - 0.014), except for change in anxiety at 12 months which was not significant (P = 0.1). Effect size was large (> 0.8) for doing regular daily tasks, pain and mobility. An intermediate ES (0.64) was seen for self-care.

**Table 2:** Outcome variables at baseline, 3 months and 12 months after surgery

Outcome variable	Baseline Mean (95% CI)_§_	3 months Mean (95% CI)_§_	12 months Mean (95% CI)_§_	P value*
**EQ-5D**_‡_**, total score**	0.32 (0.26 - 0.38)	0.63 (0.57 - 0.68)	0.60 (0.53 - 0.66)	< 0.001
**EQ-VAS**_†_**, current health**	48.0 (44.1 - 51.9)	62.0 (58.5 - 65.6)	60.4 (55.6 - 65.2)	< 0.001
**Oswestry Disability Index**	44.2 (40.6 - 47.9)	25.6 (21.9 - 29.3)	27.9 (23.4 - 32.5)	< 0.001
**VAS, back pain**	55.7 (50.5 - 60.9)	30.0 (25.0 - 35.0)	35.9 (29.3 - 42.4)	< 0.001
**VAS, leg pain**	60.2 (55.2 - 65.2)	26.4 (20.7 - 32.0)	33.6 (27-0 - 40.1)	< 0.001

*P values for all outcome variables at both time intervals were P < 0.001. _§_CI; confidence interval. _†_VAS; visual analogue scale. _‡_EQ-5D; EuroQol-5 D, instrument for assessing health related quality of life

### Secondary outcomes

#### Oswestry disability index

Mean ODI score was 44.2 at baseline, 25.6 at 3 months and 27.9 at 12 months (table 2), indicating statistically significant (P < 0.001) improvements after surgery.

#### Visual analogue scale

Mean EQ-VAS at baseline was 48.0 and this improved to 62.0 and 60.4 at 3 and 12 months respectively. Mean VAS score for back pain was 55.7, 30.0 and 35.9 at baseline, 3 months and 12 months, respectively. The mean VAS score for leg pain 60.2, 26.4 and 33.6 at baseline, 3 months and 12 months, respectively (table 2). Analyses for differences from baseline in EQ-VAS and VAS score for leg and back pain showed significant improvement after surgery (P < 0.001) at 3 and 12 months.

#### Benefits of the operation

Patients stating that the surgery had been beneficial were 82 (89.1%) at 3 and 73 (86.9%) at 12 months. Patients stating that the surgery had been very beneficial (not including "some benefits") were 59 (60.8%) at 3 and 47 (56.0%) at 12 months.

#### Walking capability

At baseline the median walking capability was 3 (able to walk up to 100 meters) and both after 3 and 12 months from baseline the median was 1 (able to walk up to 1,5 kilometers) (P < 0.001).

#### Complications

We registered a total of 18 (18.0%) complications during follow-up (table 3). The complication rate among patients above the median age (11/56, 19%) was not statistically different from those below (7/44, 16%) (P = 0.63). Three patients died during one year follow-up, but there were no inpatients deaths. One patient (1.0%) died within 3 months (26 days after surgery) of acute myocardial infarction, whereas the 2 other deaths were due to cerebrovascular accident and pneumonia at 9 and 11 months after the procedure. All non responders at 12 months (n = 19) were alive 12 months post-operative. There were 2 (2%) patients who had deep postoperative wound infections. A 72 year old man was treated with a wound revision two weeks after the operation and antibiotics for 4 weeks. The other patient was a 70 year old man who had a wound revision three weeks postoperative and he was treated with antibiotics for 5 weeks. Of the minor complications intraoperative dural tear was the most common, occurring in 9 (9%) of the patients. None required re-operation for persistent CSF leak. Unfortunately, the need for transfusions was not registered systematically. In addition, 2 (2%) patients were re-operated during the one year follow-up. The first patient was a 76 year old male who was treated with a level L3-L5 laminectomy, and he was re-operated at level L4-L5 after 4 months. He stated he had benefited very much from the operation at 12 months. The other was an 84 year old male who had bilateral recess stenosis L4-L5 and had a laminectomy as an index operation. He was re-operated with a new decompression at the same level with the same indication after 7 months, and stated that he had some benefit from the operation at 12 months.

**Table 3:** Complications type and rates

Complication type	No./total responses*
**All complications**	18/100
**Minor complications**	12/100
**Dural tear**	9/100
**Superficial wound infection**	3/100
**Major complications**	6/100
**Perioperative death within 30 days**	1/100
**Upper UTI**_†_	1/100
**Deep wound infection**	2/100
**Myocardial infarction (non-fatal)**	1/100
**Gastric ulcer**	1/100

* One missing value. _† _UTI; urinary tract infection.

#### Risk factor analyses

Patients with longer duration of leg pain had less improvement in ODI (P < 0.001, beta 0,461). A previous study indicates that leg pain more than 8 months correlates with unfavorable outcome, and we dichotomized the variable accordingly [17]. There was no significant difference between these groups (P = 0.400) with patients having symptoms less than 8 months improving 20.1 in ODI score at 12 months compared to 15.5 in patients with long standing leg pain. Patients experiencing complications had no significant difference in any outcomes (P-values between 0.277 - 0.984). The mean improvement in EQ-5 D score was 0.27 in patients with complications versus 0.31 in patients without complication. For ODI score there was a mean improvement of 17.6 in patients with complications compared to 16.8 in those without. At one year 85.7% of patients with complications stated that the operation had been beneficial compared to 87.1% of patients without complications. Patients with complications had a median hospital stay of 7 days compared to 6 days for those without complications (P = 0.215, Mann-Whitney U test due to skewed data). Increased age did not predict a worse outcome in any of the outcome variables. No other significant predictors were found for any of the outcome variables.

## Discussion

Our results demonstrate that conventional lumbar laminectomy without fusion is a safe treatment for spinal stenosis in patients 70 years and older. There were no differences in outcomes between patients who experienced complications and those who did not. Unselected patients recruited from general clinical practice can expect statistically significant improvement in health related quality of life, functional status and pain. Patients' statements about their benefit of the operation and the calculated effect size on quality of life indicate that these improvements are clinically meaningful. Our results support results from Fredman *et al *and Ragab *et al *that within an elderly population increased age is not a predictor of worse outcome [7,8].

We used the EQ-5 D score to assess health related quality of life. It has shown good reliability and proved useful for monitoring outcome of patients undergoing low-back surgery [12]. At baseline mean EQ-5 D index score was 0.32 which is lower compared to a similar age-group of the general Swedish population (0.79) [18], which is expected to be comparable to a Norwegian population due to similarities between the countries with respect to genetics, demography, socio-economic and health care system. Disease specific mean EQ-5 D index score in patients between 20-88 years suffering low back pain in Sweden is 0.66 [18], which is slightly better than the mean score after surgery in our material (0.60). We believe this, at least partly, can be explained by the fact that our patients had longstanding chronic low back problems (median duration of symptoms of 100 weeks) and 29 (28.7%) of our patients had ASA score of greater than 2, indicating severe co- morbidity. Baseline EQ-5 D and improvement after surgery is comparable to what Jansson *et al *demonstrated recently in a Swedish cohort undergoing decompressive surgery for spinal stenosis, with or without fusion, in a slightly younger population [19].

The ODI at baseline was comparable to what others have reported [15,20,21] Mean change in ODI in the present study was 5.1 points less at one year than in surgically treated patients in the SPORT trial from Weinstein *et al*, although comparable to results from previous trials [4,20,22]

In this population 70 years and older there does not seem to be an additional age-effect with respect to outcome or complications. Here we report complications occurring at comparable rates with other trials including elderly patients, although different study design and methods for registration makes direct comparison difficult [7-9,23]. In our study, patients above median age did not have worse outcomes or more complications compared to patients below the median age.

Whether type of surgery is a predictor for outcome remains controversial [1,21,24-27]. A recent study looking at trends in elderly undergoing spinal surgery for lumbar spinal stenosis reported increased use of complex fusions [28]. Compared to Rosen *et al*, using minimal invasive technique, we report higher major complication rates (6% vs 0%) but less overall complications (18% vs 39%). Also, we report slightly (4.7 points) less improvement in ODI, although their follow-up time was somewhat shorter [21]. Glassman *et al *demonstrated a good clinical outcome, but more complications, in patients 65 years or older treated with single-level lumbar arthrodesis [25]. Other studies have demonstrated that fusion procedures were associated with increased risk of major complications and death compared to decompression alone [28,29]. These findings could indicate that minimal invasive decompression might be a treamtnet as effective, and probably safer, compared to conventional laminectomy with or without fusion.

The only predictor found was that longer duration of radiating pain in the leg(s) predicted less improvement in functional status measured with changes in ODI. Although, it is not known for how long patients should try conservative treatment for lumbar spinal stenosis or when the prognosis for a favorable outcome starts to decline. Studies in patients with sciatica indicate unfavorable outcome if pain has lasted longer than 6-8 months, but we did not reproduce this finding in an elderly population with lumbar spinal stenosis [17,30]. Future studies might help us to identify the best timing of surgery.

Since our patients represent a consecutive sample from regular practice at a clinic where laminectomies are performed by several surgeons with different level of experience, we expect the external validity of our study to be good, and the data would be suitable for risk factor analyses. Although, the surgeons selected patients for surgery as part of regular practice at our institution, and this selection at doctors' discretion could lead to selection bias. Complications were prospectively assessed at 3 and 12 months - and not at discharge from the hospital or in retrospect by chart reviews. This may reduce sensitivity for minor complications, but is sensitive for delayed and major complications. The study was not designed to assess the efficacy of the surgical treatment, but it indicates that the effectiveness is acceptable. Weaknesses in our study is a relative short follow-up with for evaluating HRQL, functional status and pain - but long enough to assess safety. We used outcomes after re-operations in the 7 patients that were registered more than once in the study period, this could lead to biased results in favor of surgical treatment. Due to a small sample size it is difficult to make any meaningful conclusion regarding mortality.

## Conclusions

Properly selected patients of 70 years and older can expect a clinically meaningful improvement of HRQL, functional status and pain after decompressive laminectomy without fusion. The treatment seems to be safe. However, patients with longstanding leg-pain prior to operation are less likely to improve one year after surgery. Increased age per se should not be a contraindication for surgery.

## Competing interests

The authors declare that they have no competing interests.

## Authors' contributions

All authors read and approved the final manuscript. **ASJ: **collection of data, statistics and writing. **AS: **collection of data and writing. **SG: **statistics and writing. **ØPN: **writing and supervision. **SL: **statistics and writing. **TS: **collection of data, writing and supervision

## Pre-publication history

The pre-publication history for this paper can be accessed here:

http://www.biomedcentral.com/1471-2482/10/34/prepub
